# Cost-effectiveness evaluation of the 10-valent pneumococcal non-typeable *Haemophilus influenzae* protein D conjugate vaccine for children in Taiwan

**DOI:** 10.1186/s12962-020-00225-9

**Published:** 2020-08-28

**Authors:** Chun-Yi Lu, Ching-Hu Chung, Li-Min Huang, Eliza Kruger, Seng-Chuen Tan, Xu-Hao Zhang, Nan-Chang Chiu

**Affiliations:** 1 National Taiwan University Children’s Hospital, Taipei, Taiwan; 2 Mackay Medical College, Taipei, Taiwan; 3 IQVIA Inc, Singapore, Singapore; 4 GSK, Singapore, Singapore; 5 Mackay Children’s Hospital, No. 92, Sec. 2, Zhongshan N. Rd, Taipei City, 10449 Taiwan

**Keywords:** Pneumococcal conjugate vaccines, Taiwan, Synflorix, Prevenar 13, Invasive pneumococcal disease, Acute otitis media, Cost-effectiveness analysis

## Abstract

**Background:**

*Streptococcus pneumoniae (S. pneumoniae)* and non-typeable *Haemophilus influenzae* (NT*Hi*) are substantial contributors to morbidity and mortality of diseases including invasive pneumococcal diseases (IPDs), pneumonia and acute otitis media (AOM) worldwide. In Taiwan, 10-valent pneumococcal polysaccharide and NT*Hi* protein D conjugate vaccine (PHiD-CV) and 13-valent pneumococcal conjugate vaccine (PCV13) are licensed in children against pneumococcal disease. In addition to *S. pneumoniae*, clinical trials suggest efficacy of PHiD-CV against NT*Hi* AOM. This study aims at evaluating the cost-effectiveness of a 2 + 1 schedule of PHiD-CV vs. PCV13 2 + 1 in the universal mass vaccination program of infants in Taiwan.

**Methods:**

A published Markov cohort model was adapted to simulate the epidemiological burden of IPD, pneumonia and AOM for a birth cohort in Taiwan over 10 years. The probability of entering a specific health state was based on the incidence rate of the diseases. Only direct medical costs were included, and costs and outcomes were discounted annually. Vaccine efficacy assumptions were based on published data and validated by a panel of independent experts. Clinical, epidemiological, and serotype distribution data were based on locally published data or the National Health Insurance Research Database. Price parity of vaccines was assumed. Published pneumococcal disease-related disutility weights were used due to lack of local data. Incremental cost-effectiveness ratio was calculated and benchmarked against the recommended threshold in Taiwan. Extensive one-way sensitivity analysis, alternative scenarios and probabilistic sensitivity analysis were performed to test the robustness of the results.

**Results:**

PHiD-CV would potentially reduce the number of NT*Hi*-related AOM cases substantially and prevent comparable IPD and pneumonia-related cases and deaths compared to PCV13. Over a 10-year horizon, PHiD-CV is estimated to dominate PCV13, saving 6.7 million New Taiwan Dollars (NTD) and saving 21 quality-adjusted life years. The result was robust over a wide range of sensitivity analyses. The dominance of PHiD-CV was demonstrated in 90.5% of the simulations.

**Conclusions:**

PHiD-CV 2 + 1 would provide comparable prevention of IPD, pneumonia cases and additional reduction of NT*Hi*-AOM cases, and is considered dominant compared with PCV13 2 + 1 in Taiwan.

## Background

*Streptococcus pneumoniae (S. pneumoniae)* is an important cause of a spectrum of diseases worldwide, and can cause invasive pneumococcal diseases (IPDs) including meningitis and bacteraemia, non-invasive lower respiratory tract infections such as pneumonia, and non-invasive upper respiratory tract infections, which include sinusitis and acute otitis media (AOM).

*Haemophilus influenzae (H. influenzae)* is another major driver of infection, particularly in young children [1]. Non-typeable *Haemophilus influenzae* (NT*Hi*), is most commonly linked with mucosal diseases, such as otitis media and sinusitis [2]. Over the years, the prevalence of NT*Hi* infections has increased, while at the same time *H. influenzae* type b disease has relatively decreased with the introduction of routine immunization of children, IPDs affect people of all ages, with the greatest burden of disease among young children and older adults, although the incidence varies country-to-country and over time. In Taiwan, it is mandatory to report IPD cases within 7 days of establishing a laboratory-confirmed diagnosis and provide isolated *S. pneumoniae* to the Taiwan Center for Disease Control (TCDC). Between 2008 and 2013, there were 4453 cases of IPD reported, with peak incidences amongst adults > 75 years and children 2–4 years [3, 4]. This is atypical compared with the usual epidemiology pattern of peak incidences from other countries, with the highest incidence rate generally seen in children < 2 years [5–7]. Taiwan has observed serotype replacement in IPD, notably the increased prevalence of serotype 19A, with the proportion of serotype 19A for all ages increasing from 5.5 to 25.3% from 2008 to 2012 [3, 4]. In Taiwan, AOM has caused significant health and economic burden for children < 5 years, with greater than 200,000 cases per year [8]. *S. pneumoniae* and NT*Hi* infections were identified as the top two bacterial pathogens that cause AOM worldwide. A retrospective study of paediatric patients with culture-proven AOM in a hospital in Taiwan found that between 1999 and 2008 the most commonly isolated pathogens were *S. pneumoniae* (68%) followed by NT*Hi* (19%) [9]. Over the time period, there was a progressive reduction in the number of patients with AOM caused by the *S. pneumoniae* bacterium, which might be due to a couple of reasons such as antibiotics prescription changes and vaccination [9].

At present, two pneumococcal conjugate vaccines are licensed to vaccinate children in Taiwan. Synflorix (GSK), a pneumococcal polysaccharide and NT*Hi* protein D conjugate vaccine (PHiD-CV) and Prevenar 13 (Pfizer), a pneumococcal 13-valent conjugate vaccine (PCV13). In children less than 5 years of age, both vaccines are licensed locally for active immunization against diseases caused by *S. pneumoniae* (including meningitis, sepsis, bacteraemia, pneumonia, and AOM). In addition, PHiD-CV has published efficacy trial data to suggest efficacy against AOM caused by NT*Hi* with the current formulation and a previous 11-valent formulation [10, 11].

To combat pneumococcal diseases among the paediatric population in Taiwan, the government introduced a paediatric pneumococcal conjugate vaccination program in 2013. A one-dose PCV13 catch-up universal mass vaccination (UMV) was first implemented in March 2013 for children aged 2–5 years and then expanded to two-dose PCV13 for children aged 1–5 years from January 2014 onwards. To further reduce the clinical and economic burden of pneumococcal diseases for new birth cohorts, the government has decided to implement PCV13 2 + 1 UMV program (at 2, 4, and 12 months) in primary birth cohorts from 2015 onwards.

Health economic assessments have been increasingly incorporated into the comprehensive health technology assessment for vaccine policy decision-making over the past decade and more recently within the Asia–Pacific region. In Taiwan, local health economic submission is now mandated for a vaccine to be qualified for UMV tenders. A previous study was conducted by Chang et al. [8] to evaluate the burden of pneumococcal diseases in Taiwan from 2002–2008. Due to the changing epidemiology of pneumococcal diseases in Taiwan [3], and the availability of new effectiveness data of the vaccines [11–14], there is a need to update the cost-effectiveness analysis with the latest data to inform decision-making on the choice of vaccine for the upcoming UMV in Taiwan.

The objective of this study is to evaluate the cost-effectiveness of a UMV program with a 2 + 1 schedule of PHiD-CV vs. a 2 + 1 schedule of PCV13 in Taiwan.

## Methods

### Markov model

**Fig. 1:**
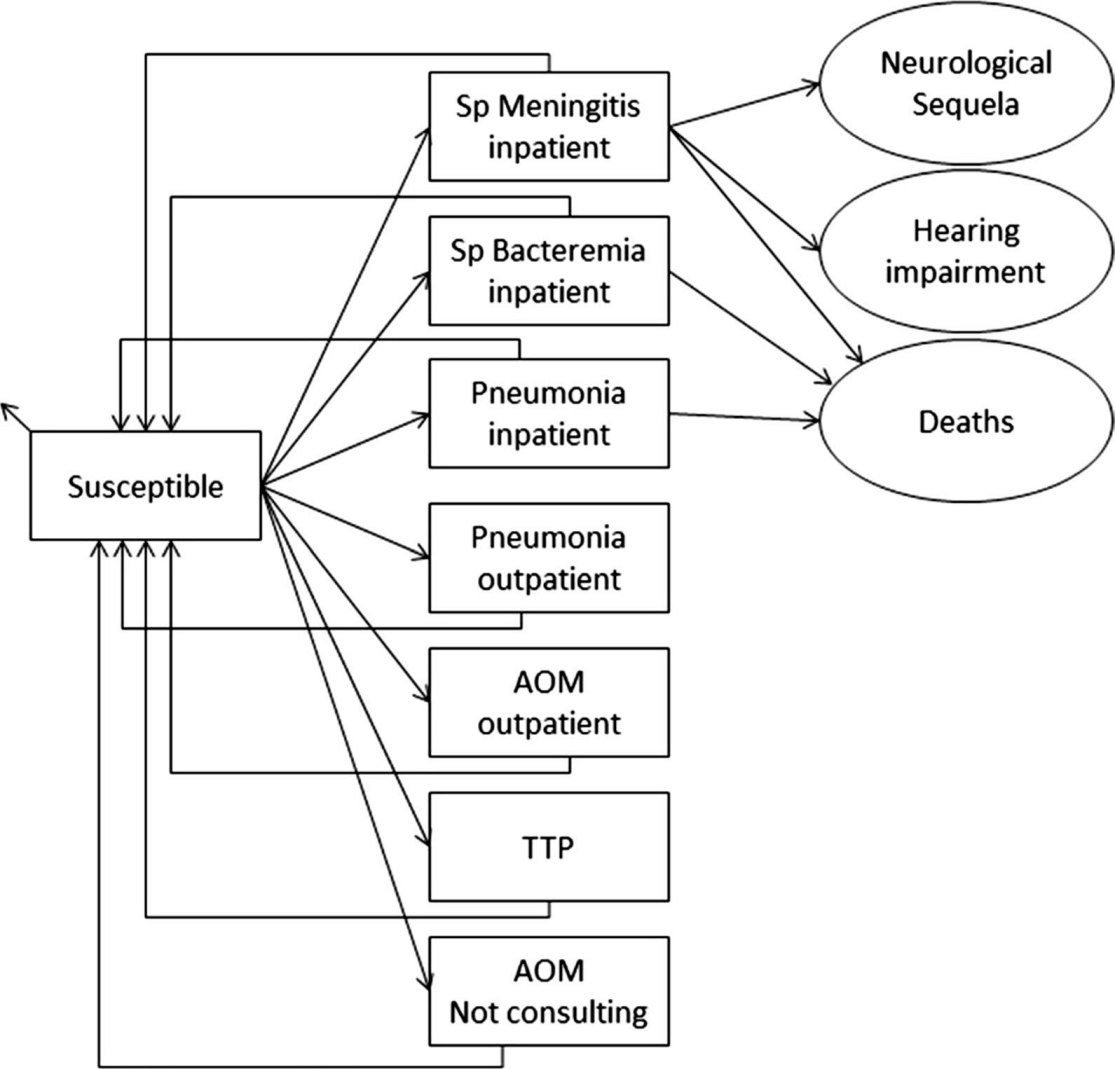
Markov cohort model design. Rectangles represent mutually exclusive health states. Age-specific incidences are applied monthly to the susceptible population. Circles (sequelae and death) and small arrows (natural death) represent the proportion of the population removed from the model. Costs and benefits are computed monthly and aggregated over the cohort's lifetime. Non-consulting AOM episodes are accounted for in the quality-of-life calculation. Sp: Streptococcus pneumoniae; AOM: Acute Otitis Media; TTP: Tympanostomy Tube Placement

A published Markov cohort model was adapted to simulate the epidemiological burden of pneumococcal and NT*Hi*-related diseases, including IPDs, pneumonia and AOM, within a registered live birth cohort in Taiwan [15, 16]. The cohort-based analyses are commonly used for health economic modelling for determining the direct impact of medical interventions [16], and the current model has been used by previous cost effectiveness studies in other countries [17–19]. Figure 1 shows the Markov cohort model for the analyses. In this model, the individuals of the birth cohort were simulated to move between the Markov states according to estimated transition probabilities. The model has a number of mutually exclusive disease-related outcomes including IPDs, pneumonia, AOM, no pneumococcal infection, and death. During each cycle, the probability of entering a specific health state was calculated using the incidence rates of the diseases.

In the base case, the birth cohort was followed for 10 years from birth, with a lifetime time horizon used as an alternative scenario. A 10-year time horizon was chosen for the base case for two reasons; (1) serotype distribution is expected to be changing over time in the country post introduction of a pneumococcal conjugate vaccine [20] and; (2) the efficacy of the vaccine(s) was assumed to last for approximately 10 years [21].

For both options (vaccination with PHiD-CV or vaccination with PCV13), the model estimated the expected effect of vaccination for each of the disease states. Costs and quality-adjusted life years (QALYs) specific to each health state were estimated from birth to 10 years of age and the total accumulated costs and QALYs calculated. Costs and outcomes were discounted by 3% in the base case. Incremental cost-effectiveness ratios (ICERs) were computed, comparing the marginal benefits and costs of PHiD-CV vs. the PCV13. We benchmarked the results based on local pharmacoeconomic guidelines, whereby cost-saving is described as a strategy with lower costs and higher QALYs, a cost-effective intervention is one in which the ICER is between 1 and 3 Gross Domestic Product (GDP) per capita of the country and an intervention is not cost-effective if the ICER is over 3 GDP per capita of the country (GDP per capita in 2012 in Taiwan = 631,142 NTD) [22, 23].

### Epidemiological data

The birth cohort size utilized in the analysis was 229,481 newborns (in 2012). We accessed the National Health Insurance Research Database (NHIRD) of the 2012 entire population records to evaluate both incidence and direct medical costs of IPDs, all-cause pneumonia and AOM, with approval from the Ministry of Health and Welfare.

Consecutive records of the same patient with a 30-day interval between discharge and re-entry were defined as the same episode of disease for inpatient and outpatient visits. The ICD-9 codes used to identify incidence are included in the supplementary materials section. However, local clinical experts considered the incidence data for IPD retrieved from the NHIRD to be a significant underestimation, as most age groups did not have any cases and the number of cases was very different from the published Taiwan surveillance data [8, 24]. Therefore, local experts agreed to use the local surveillance data for both incidence data and IPD serotype distribution data [3, 24].

After validation by local experts, age-specified NHIRD data were used for the incidence of inpatient and outpatient cases for both all-cause pneumonia and AOM. Case-fatality rates (CFR) were based on the 2002–2007 unpublished data on the disease burden of pneumococcal diseases in Taiwan by Chang et al. [25]. Due to the lack of precise ICD-9 codes to capture the long-term sequelae related to IPD, it was decided not to include long-term sequelae in our analysis. A summary of the epidemiological model parameters is available in Table 1.

**Table 1:** Taiwan model parameters

Parameter	Value
Birth cohort (2012)	N = 229 481
Serotype distribution	Age-specific data; see Additional file 1: Table ST4
IPD	
1. Incidence rate (per 100,000)	Age-specific data; see Additional file 1: Table ST1
2. CFR (%)	Age-specific data; see Additional file 1: Table ST1
All-cause pneumonia	
1. Hospitalization rate (per 100,000)	Age-specific data; see Additional file 1: Table ST2
2. CFR (%)	Age-specific data; see Additional file 1: Table ST2
3. GP consultation rate (per 100,000)	Age-specific data; see Additional file 1: Table ST2
AOM	
1. GP consultation rate < 5 y (per 100,000)	Age-specific data; see Additional file 1: Table ST3
2. Hospitalization rate (per 100,000)	Age-specific data; see Additional file 1: Table ST3
Discount rate (per annum)	3%

*AOM* acute otitis media, *CFR* case-fatality rate, *IPD* invasive pneumococcal disease, *GP* general practitioner, *y* year

### Vaccine effectiveness

#### IPD effectiveness

This model assumed a ramp-up protection for the 2 + 1 regimen (with 50, 90 and 100% of the assumed effectiveness attained with each of the 3 doses administered, respectively). We assumed that full efficacy would be achieved after the final dose and that vaccine efficacy (VE) would begin to decline at the age of 3, deteriorating to zero by the age of 10. In the base case, we assumed no herd effect. VE against IPD was calculated as a sum-product of local serotype distribution (based on the latest published IPD surveillance data in Taiwan) and serotype-specific efficacy of each vaccine [3, 24]. IPD serotype distribution in Taiwan is available in Additional file 1: Table ST4.

Randomized controlled trial data are not available on the efficacy of PCV13 against IPD in children; however, real-world effectiveness data are available. On the other hand, the efficacy of PHiD-CV against vaccine-type (VT) IPD has been demonstrated in two double blind randomized controlled trials, one conducted in Finland (FinIP) [92% (95% confidence interval (CI): 58–100%)] for 2 + 1 dosing schedule; [100% (95% CI: 83–100%)] for 3 + 1 dosing schedule) [26]; and the second conducted in Latin America (Clinical Otitis Media & Pneumonia Study (COMPAS) [100% (95% CI: 77–100%)] for 3 + 1 dosing schedule) [11]. Further, post-marketing surveillance has shown both PHiD-CV and PCV13 reduce the incidence of VT IPD [12, 27]. However, the evidence of serotype-specific effectiveness for PHiD-CV and PCV13 for the majority of the individual serotypes was limited due to the low incidence of the seven serotypes common to the two vaccines and PCV7 as many countries had a prior mass vaccination programs using the latter. Therefore, the serotype-specific effectiveness data were largely extrapolated from estimates of VE developed from a CDC case–control study conducted in the US for PCV7 and reported by Whitney et al. [28].

It was assumed that the 10 common types covered by both PHiD-CV and PCV13 (1,4,5,6B,7F,9V,14,18C,19F,23F) would have an efficacy of 94.7%, which is the mean of the serotype-specific estimates (≥ 1 dose) for the 7 serotypes included in PCV7 (Table 2).

**Table 2:** Vaccine effectiveness against all-cause pneumonia, IPD and AOM

Serotype	PHiD-CV	PCV13	References
All-cause pneumonia			
Inpatient	23.4%	23.7%	[11]
Outpatient	7.3%	7.3% (assumed to be the same as PHiD-CV)	[11]
IPD			
VT (10 common ST)	94.7%	94.7%	[28]
3	0%	0%	[32] [33]
6A	76.0% (assumed to be the same as PCV7)	94.7% (VT)	[28]
19A	71% (base case) 19% (used in scenario analysis: worst case using Taiwan point estimate of PCV7/10)	80%	[12, 13, 34]
AOM			
AOM VT VE 10 common serotypes	69.9%	69.9%	[11]
ST3 AOM VE	0% [10]	0%	[11]
6A AOM VE	29.0%	69.9%	[11]
19A	29.0%	69.9%	[11]
NVT AOM VE	−33%	−33%	[35]
NT*Hi* AOM VE	21.5% [11] [0–35.3% in sensitivity analysis]	0% (based on expert opinion) [−11 to + 8% in sensitivity analysis]	[11, 35, 36]
AOM Myringotomy	50.92%	30.6%	Estimated based on [37, 38] and overall AOM efficacies above

*AOM* acute otitis media, *IPD* invasive pneumococcal disease, *NTHi* non-typeable *Haemophilus Influenzae*, *NVT* non-vaccine type, *PCV7/13* 7/13-valent pneumococcal conjugate vaccine, *PHiD-CV* 10-valent pneumococcal polysaccharide and NT*Hi* protein D conjugate vaccine, *ST* serotype, *VE* vaccine effectiveness, *VT* vaccine-type

Evidence for cross-protection for PCV7 against serotype 6A (through the inclusion of cross-reactive 6B serotype) has been demonstrated in many countries [28, 29]. PHiD-CV, which also contains serotype 6B, was assessed to be immunologically non-inferior to PCV7 [30], and real-world protection was observed from PHiD-CV use in a UMV program in Finland [14]. It should be noted that prior PCV7 use in many countries has reduced the incidence of IPD caused by serotype 6A significantly and hence, the confidence intervals around impact studies assessing subsequent PHiD-CV use can be wide. Based on the above evidence, cross- protection for 6A of PHiD-CV was assumed at 76% [14, 28, 31].

PHiD-CV, based on recently published evidence from various countries, has been indicated for IPD caused by 19A in Taiwan in July 2018 [39]. Real-word effectiveness data from post-marketing case–control studies in Quebec [VE 71% (95% CI: 24–89%)] [12], and Brazil [VE 82% (95% CI: 11–96%)] [13], a population-based study in Finland [VE 62% (95% CI: 20–85%)] [14], and a surveillance study from the Netherlands [VE 62% (95% CI: 33–81%)] [40], have all demonstrated significant impact of PHiD-CV on 19A IPD. The case–control study from Quebec, Canada additionally demonstrated no substantial difference in effectiveness against 19A IPD between PHiD-CV and PCV13 [71% (95% CI: 24–89%) vs. 74% (95% CI: 11–92%), respectively, p-value > 0.05]. Consequently, a VE value of 71% was assumed for PHiD-CV in the base-case scenario as per latest Quebec data and a range of 19–82% used in the sensitivity and scenario analyses [34]. The lower estimate of 19% was chosen as it was the lowest effectiveness observed for children receiving either PCV7 or PCV10 against 19A IPD (in this instance, IPD occurring after the age of 2 years) as reported from a Taiwanese effectiveness study. The upper estimate of 82% was the effectiveness reported from the Brazilian case–control study [13].

Real-world evidence on the effectiveness of PCV13 on 19A IPD have shown that the estimates are usually lower than the estimates for the serotypes shared with PCV7 [12, 33, 41, 42]. Subsequently, an estimate of 80% was used for PCV13 (the highest reported VE of PCV13 3 + 1 schedule from the US was 86% [41]; the Quebec data on PCV13 2 + 1 were 74% [12]; local experts suggested to use 80% in the base-case analysis as an optimal assumption) [12, 41].

There is conflicting evidence on the effectiveness of PCV13 for protecting against serotype 3 IPD infections [27, 33, 43–46]. Based on recent data from the UK for a 2 + 1 schedule, a statistically non-significant effectiveness estimate of 26% (95% CI: −69–68%) was observed for PCV13 against serotype 3 [33]. This lack of effectiveness of PCV13 against serotype 3 was highlighted by the UK Joint Committee on Vaccination and Immunisation (JCVI) [47]. In this analysis, an effectiveness of 0% was assumed in the base case and a value of 26% was included in a sensitivity analysis. However, given the limited circulation of serotype 3 in the paediatric population in Taiwan, based on the latest surveillance data, this assumption had limited impact on the result in this model [3, 24].

#### All-cause pneumonia effectiveness

To date, it has been very difficult to predict with precision the relative impact of one vaccine formulation over another for pneumonia. There have been several pneumonia efficacy trials with vaccine formulations containing 7, 9, 10, and 11 serotypes [11, 48–52]. All of the studies gave efficacy point estimates against this endpoint within a range of 20–35%, with no indication that vaccines with more serotypes provided correspondingly greater protection against pneumonia (in fact the largest difference is among the studies with PCV9) [48, 50, 52]. An independent evaluation of the effectiveness of PCVs in Latin America conducted through a systematic review also concluded that there was no evidence of either of the two vaccines (PCV13 and PHiD-CV) being superior to the other [53]. Furthermore, it would be unlikely to find a significant difference favoring a vaccine with more serotypes, as pneumonia is multifactorial in terms of disease-causing pathogens, with a major proportion of disease cases caused by pathogens other than *S. pneumoniae* [54]*.*

The COMPAS study demonstrated efficacy of 23.4% (95% CI: 8.8–35.7%) for inpatient pneumonia and 7.3% (95% CI: 2.1–12.3%) for outpatient pneumonia for PHiD-CV [11]. In the absence of PCV13-specific pneumonia VE estimates, local experts agreed to use the same figure for PCV13 outpatient pneumonia at 7.3%. For inpatient pneumonia, even though there was no study showing superior efficacy of a vaccine with higher valence, the local experts, suggested allocating a higher value for PCV13 to take into consideration the proportion of pneumonia cases due to 19A (approximately 12% of the all-cause pneumonia cases were estimated to be due to 19A infection). For PCV13, this proportion of all-cause pneumonia was assumed to have a proportionally higher effectiveness based on the effectiveness values assumed for 19A IPD for PHiD-CV and PCV13 (71% vs. 80%, respectively)—or a 13% higher efficacy. Therefore, the effectiveness estimate of PCV13 against all-cause pneumonia was 23.8% (Table 3).

**Table 3:** Estimation of effectiveness against all-cause pneumonia for PCV13

	Proportion of all-cause pneumonia (%)	Assumed VE
19A	12%	23.4% × 1.13^‡^
All other pneumonia	88%	23.4%
(Weighted) Total		*23.8%*
^‡^ refer to calculation below		
	PHiD-CV	PCV13
VE 19A IPD	71%	80%
VE ratio, (PCV13:PHiD-CV)	1.13	

^‡^Calculation of 1.13 as the VE ratio

*IPD* invasive pneumococcal disease, *PCV13* 13-valent pneumococcal conjugate vaccine, *PHiD-CV* 10-valent pneumococcal polysaccharide and NT*Hi* protein D conjugate vaccine, *VE* vaccine effectiveness

#### AOM effectiveness

The overall efficacy of PHiD-CV against AOM has been demonstrated in the latest double-blinded randomized controlled trial [11]. So far, there are insufficient randomized controlled trial (RCT) data available on the efficacy of PCV13 against overall or pathogen-specific AOM. Based on the literature review, the bacterial causes of AOM have remained largely the same for the past half-century [55]. However, NT*Hi* has become a more important or even the dominant pathogen in recent years, potentially due to replacement issues reported with PCV7 [35, 36, 56–58]. Therefore, VE against AOM was estimated based on efficacy against pneumococcal vaccine serotypes and non-vaccine serotype diseases and efficacy against diseases caused by NT*Hi*.

For the base-case scenario, the weighted averages of AOM pathogen distribution due to *S. pneumoniae* and NT*Hi* across 23 different datasets of different countries were used (35.9% for *S. pneumoniae* and 32.3% for NT*Hi*) [55]. In the sensitivity analysis, a retrospective local AOM etiology study by Kung et al. [59] was used, which reported higher *S. pneumoniae* (55.7%) and lower NT*Hi* prevalence (22.9%). This distribution was selected as a sensitivity analysis since it only included moderate to severe AOM, including cases requiring tympanocentesis, which is not routinely performed in mild cases in Taiwan [59]. Due to the lack of better local data, the experts advised the use of data from Kung et al. [59] for the estimation of the pneumococcal serotype distribution of AOM cases, though the sample size was very small, with only 39 episodes reported in the study.

VE against all cause clinical AOM was calculated from VE against (i) pneumococcal vaccine serotypes and non-vaccine serotypes and (ii) AOM caused by NT*Hi*. VE against AOM caused by pneumococcal vaccine serotypes was assumed to be 69.9% (95% CI: 29.8–87.1) based on the observations from the COMPAS trial, a randomized controlled trial of PHiD-CV. VE against pneumococcal non-vaccine types was estimated to be −33% based on the FinOM study to account for serotype replacement. VE against NT*Hi* AOM was assumed to be 21.5% (95% CI: −43.4–57.0) based on the COMPAS trial despite the trial not being powered to estimate this end-point [11]. However, this result is in line with the findings from the POET trial, which used an 11-valent formulation of PHiD-CV; VE = 35.3% (95% CI: 1.8–57.4) [10]. To be conservative, the experts advised to use the lower estimate in COMPAS and use POET figures in the sensitivity analysis.

Two studies assessed the impact of PCV7 on myringotomies/ tympanostomy tube procedures (TTP)—one RCT in the US estimated an efficacy of 23.2% (based on a TTP incidence of approximately 1.1%) [37]; another study from Finland observed a 4% reduction based on an incidence of 12.7% [38]. Fitting an exponential function between these two points results in an incidence-specific VE estimate for myringotomies with PCV7. Based on the overall AOM effectiveness calculated based on the description earlier, PCV13 is estimated to be 1.13 times more effective than PCV7. Thus, the exponential function obtained for PCV7 can be “shifted” up proportionally to obtain the relevant myringotomy efficacy curve for PCV13. A similar curve can also be obtained for PHiD-CV. The base-case estimates used in the model can then be calculated by using the obtained hospitalized myringotomy incidence for Taiwan (as described in Additional file 1: Table ST3).

Other assessments of PCV impact on AOM were not considered in this analysis because they did not provide pathogen-specific VE values that were needed to adjust the model, according to the causative AOM pathogen distributions observed in different countries or regions. Due to lack of local data, long-term sequelae were not included in this analysis.

VE of PHiD-CV against all-cause pneumonia, IPD and AOM are available in Table 2.

### Health outcomes and utilities

This model was designed to estimate the impact of diseases by including the respective QALYs lost in acute episodes. Due to the lack of pneumococcal disease(s)-related disutilities in the Taiwanese population, published disutility weights were used [16, 60–62]. Table 4 displays disutility values.

**Table 4:** Disutilities of pneumococcal diseases

Short-term	Disutility	Reference/assumptions
IPD (inpatient)	0.023	[60]
Pneumonia (inpatient)	0.008	Assumed to be the same as for inpatient bacteraemia
Pneumonia (outpatient)	0.006	Value for local infection[60]
AOM (outpatient)	0.005	[62]
AOM (inpatient)	0.005	Assumed to be the same as for acute otitis media

*AOM* acute otitis media, *IPD* invasive pneumococcal disease

### Resource use and costs

The analyses were conducted from the perspective of the Taiwanese National Health Insurance system. Therefore, only direct medical costs (e.g. hospitalization, inpatient/outpatient diagnostic tests and procedures, medication/vaccine costs, and healthcare professionals’ fees) were included. Direct medical costs data for the acute episodes were based on the retrieved 2012 NHIRD data which were provided by Health and Welfare Data Science Center and are available in Table 5. In addition, price parity of both vaccines at NTD 1269.5/dose (the current PCV13 UMV price) was applied in the base case.

**Table 5:** Costs utilized in the model

Median cost per acute episode	Children (age < 18)	Adult (age ≥ 18)
IPD—first year (acute episode)	NTD 74 226	NTD 102 488
Pneumonia—hospitalized	NTD 14 006	NTD 41 445
Pneumonia—outpatient	NTD 494	NTD 675
AOM hospitalized cases	NTD 14 246	NTD 49 848
AOM GP consultations	NTD 460	NTD 414

*AOM* acute otitis media, *GP* general practitioner, *IPD* invasive pneumococcal disease, *NTD* new Taiwan dollar

### Sensitivity analyses

Extensive one-way sensitivity analyses were performed to evaluate the robustness of the results and conclusions to changes in model variables. These were performed using ± 20% (up to ± 50% depending on the inputs) for each of the base-case value of most variables, or alternatively the upper and lower limits of the 95% CI when available. A probabilistic sensitivity analysis (PSA) was also performed using 1000 Monte Carlo simulations to assess the robustness of the base-case result.

### Scenario analyses

In addition to the sensitivity analyses, we performed additional scenario analyses to determine the effect of changing key assumptions in the model. This includes changing the efficacy of PHiD-CV against serotype 19A, reducing the AOM inpatient incidence rate, adjusting the proportion of AOM cases caused by the respective bacterium, adjusting the efficacy of PCV13 against pneumonia, reducing the efficacy of PHiD-CV against NT*Hi,* and, finally, reducing the price of PHiD-CV by 10%.

## Results

### Health outcomes and economic impact

Table 6 presents the estimated impact, in terms of health and economic outcomes, of the PHiD-CV vs. PCV13 vaccination programs for the 2012 birth cohort (n = 229,481) in Taiwan. It was projected that PHiD-CV would prevent an additional 4424 cases of AOM and allow a comparable reduction in IPD and pneumonia-related cases. There was no difference in all-cause deaths over 10 years between the two vaccines.

**Table 6:** Health outcomes and economic impact of PHiD-CV vs. PCV13 vaccination programs

	PCV13 (A)	PHiD-CV (B)	Difference (B−A)
Health outcomes			
IPD cases (acute episode)	99	108	9
All-cause pneumonia cases (acute episode)	19,714	19,754	40
AOM cases (acute episode)	135,206	130,783	−4424
Deaths due to IPD/pneumonia	17	17	0
Costs			
Vaccination	NTD 926 465 468	NTD 926 465 418	− NTD 50
Acute episode			
IPD	NTD 7 341 171	NTD 7 992 670	NTD 651 499
All-cause pneumonia	NTD 352 720 474	NTD 353 278 362	NTD 557 957
AOM	NTD 189 117 177	NTD 178 291 679	− NTD 10 825 498
Total			
Undiscounted	NTD 1 475 644 291	NTD 1 466 028 130	- NTD 9 616 161
Discounted	NTD 1 392 017 676	NTD 1 383 217 323	- NTD 8 800 353

*AOM* acute otitis media, *IPD* invasive pneumococcal disease, *PCV13* 13-valent pneumococcal conjugate vaccine, *PHiD-CV* 10-valent pneumococcal polysaccharide and NT*Hi* protein D conjugate vaccine, *NTD* new Taiwan dollar

Results showed that the total discounted savings from the PHiD-CV 2 + 1 compared to the PCV13 2 + 1 were estimated to be approximately 8.8 million NTD. The majority of these savings were due to the reduction in incidence and respective costs related to AOM.

#### Incremental cost-effectiveness ratios

Table 7 presents the results of the cost-effectiveness analysis of PHiD-CV vs. PCV13 in Taiwan. For the base-case scenario, the total discounted QALYs gained with the PHiD-CV vaccination program was projected to be 21, meaning 21 additional years in ‘perfect’ health when compared with PCV13. The total discounted savings with the PHiD-CV 2 + 1 vaccination program compared to the PCV13 program was projected at approximately 8.8 million NTD. Therefore, the PHiD-CV vaccination program was a cost-saving (or “dominant”) strategy compared with PCV13 for Taiwan. This was a conservative estimation as we did not take into account the costs associated with complications, long-term sequelae and antibiotics use commonly associated with AOM.

**Table 7:** Incremental cost-effectiveness ratio of PHiD-CV vs. PCV13 vaccination programs

	PCV13 (A)	PHiD-CV (B)	Difference (B−A)
Total discounted costs (NTD)	1 392 017 676	1 383 217 323	-8 800 353
Total discounted QALYs gained	1 799 828	1 799 849	21
Incremental cost-effectiveness ratio	Dominant (cost saving)		

*PCV13* 13-valent pneumococcal conjugate vaccine, *PHiD-CV* 10-valent pneumococcal polysaccharide and NT*Hi* protein D conjugate vaccine, *NTD* new Taiwan dollar, *QALY* quality-adjusted life year

### Sensitivity analyses

#### One-way sensitivity analyses

**Fig. 2:**
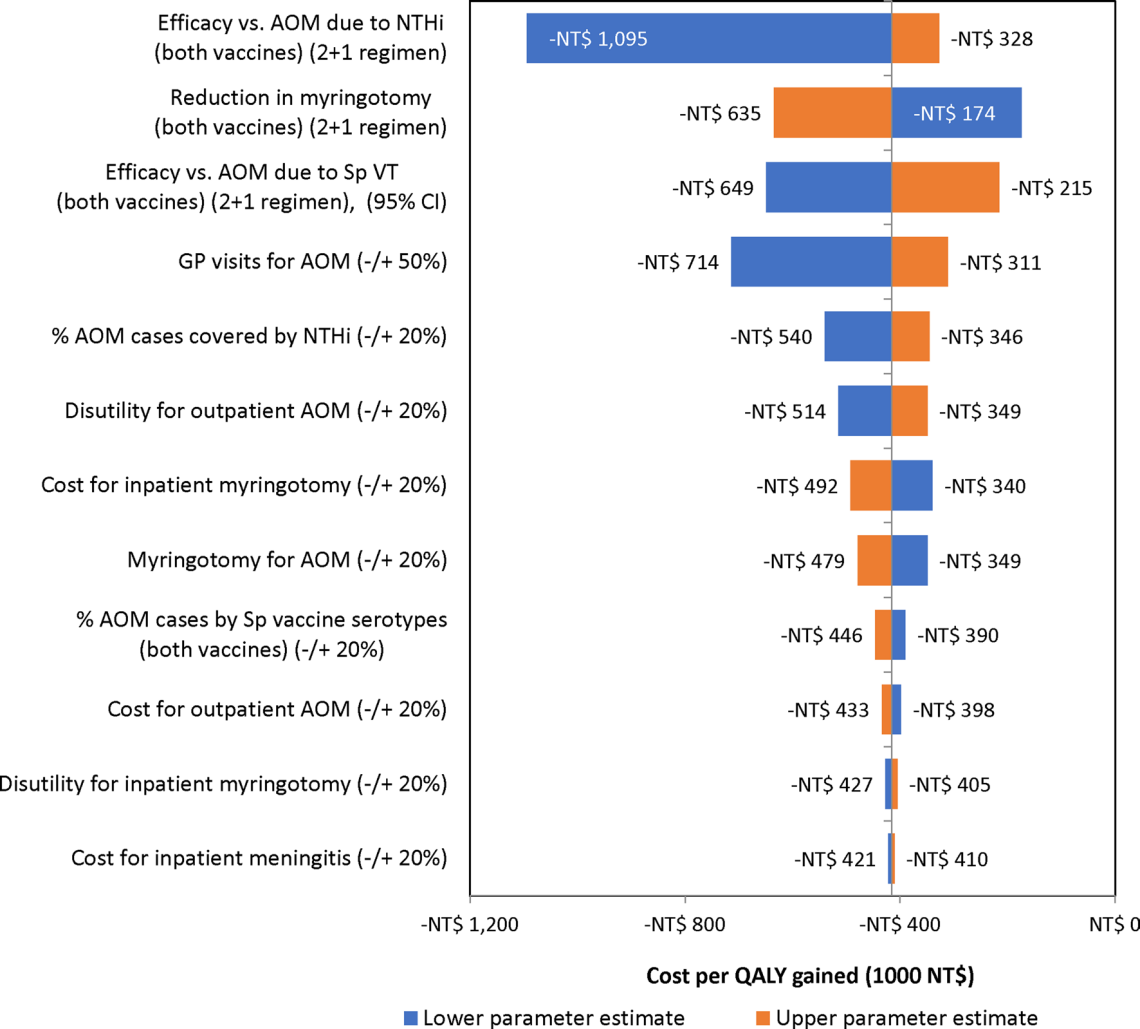
One-way sensitivity analyses tornado plot for PHiD-CV vs. PCV13. AOM: acute otitis media; GP: general practitioner; PCV13: 13-valent pneumococcal conjugate vaccine; PHiD-CV: 10-valent pneumococcal polysaccharide and NT*Hi* protein D conjugate vaccine; NT$: new Taiwan dollar; QALY: quality adjusted life year Sp: *Streptococcus pneumoniae*; VT: vaccine type

In the one-way sensitivity analyses, the cost-saving result of the PHiD-CV vaccination program vs. PCV13 was found to be very robust. As expected, the effectiveness, and epidemiological parameters around AOM are the most sensitive ones (Fig. 2). Marginal differences were assumed between the vaccines in terms of serotype-specific IPD efficacies and so too for all-cause pneumonia. Additionally, the incidence of AOM is estimated to be nearly 700 times higher than that of IPD in children < 5 years of age. Thus, the results are driven by the assumed relative efficacy estimates for both vaccines against AOM.

#### Probabilistic sensitivity analyses

**Fig. 3:**
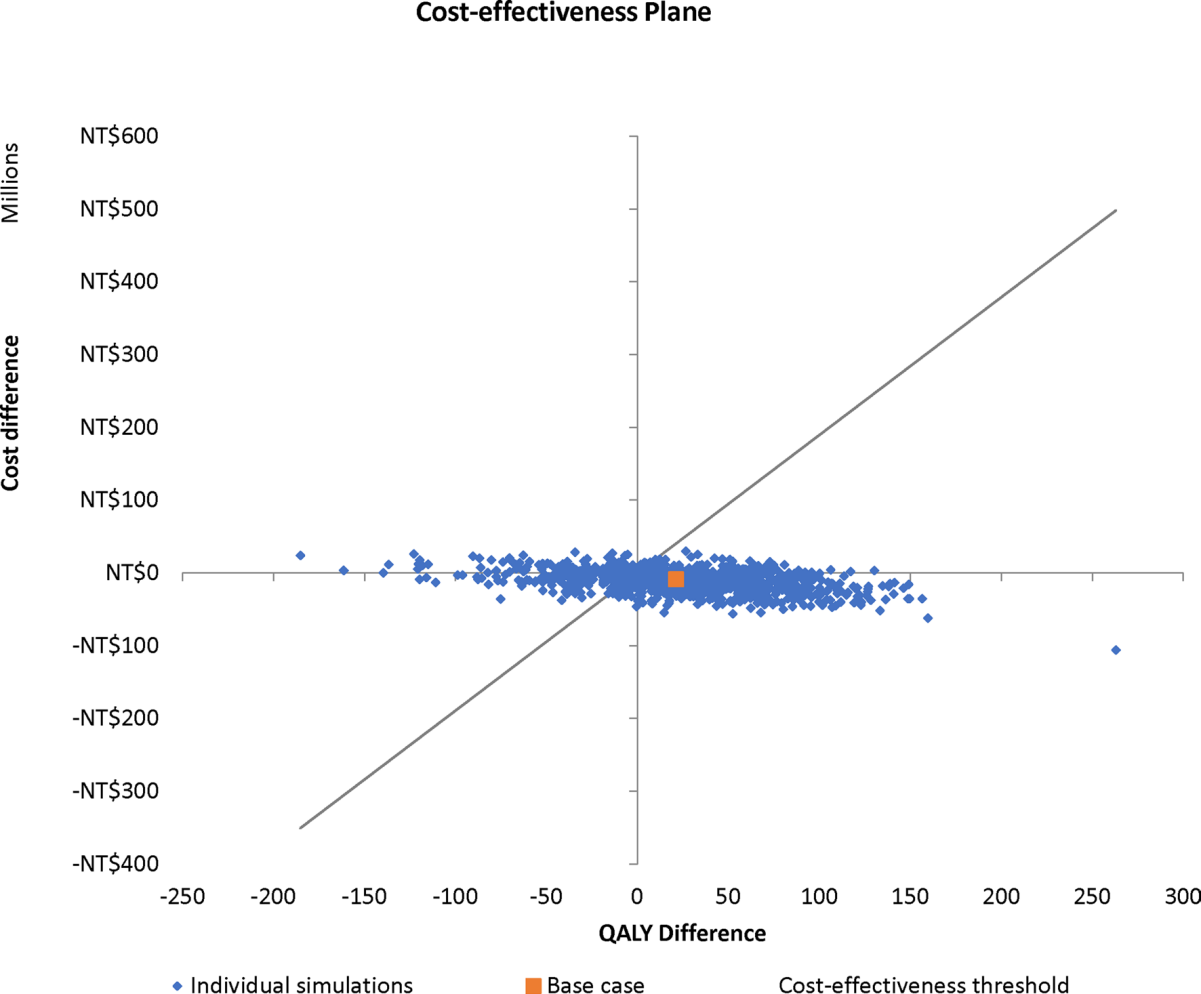
Probabilistic sensitivity analysis plot for PHiD-CV vs. PCV13. NT$: new Taiwan dollar; QALY: quality adjusted life year

A monte-carlo simulation over 1000 iterations moderately supported the robustness of the base-case result (Fig. 3). The PSA showed that at price-parity PHiD-CV was dominant compared to PCV13 (i.e., being more effective and less costly) in 61% of simulations. PCV13 dominated PHiD-CV in 12% of the simulations.

### Scenario analyses

In addition to sensitivity analyses, we performed several scenario analyses to test the cost-saving result to changes in key parameters (Table 8).

**Table 8:** Results from the scenario analyses of PHiD-CV vaccination program vs. PCV13 in Taiwan

Parameter	Base-case	Scenario analysis	Cost (millions)	QALY	ICER
Efficacy of PHiD-CV against 19A IPD	71%	19%	−0.6	8	Dominant (cost saving)
Efficacy of PCV13 against ST3 IPD	0%	26%	−6.6	21	Dominant (cost saving)
AOM inpatient IR reduced by 50% (2–9 years old) [per 100,000]	1 year: 1011.3; 2 year: 1026.1; 3 year: 826.2 4 year: 795.8; 5 year: 245.8	1 year: 505.7; 2 year: 513; 3 year: 413.1; 4 year: 397.9; 5 year: 122.9	−3.9	20	Dominant (cost saving)
% of AOM cases caused by *S. pneumoniae* vs. NT*Hi*	35.9 (*S. pneumoniae*); 32.3 (NT*Hi*) [55]	55.7 (*S. pneumoniae*); 22.9 (NT*Hi*) [59]	−0.8	6	Dominant (cost saving)
Efficacy against pneumonia	PHiD-CV: 23.4%	PHiD-CV: 23.4%	−7.1	21	Dominant (cost saving)
	PCV13: 23.7%	PCV13: 23.4%			
Efficacy of PCV13 against NT*Hi* AOM reduced to −11%	0%	−11%	−14.4	38	Dominant (cost saving)
Efficacy of PHiD-CV against NT*Hi* AOM reduced to 0	21.5%	0%	0.15	38	Dominated
Time horizon	10 years	Life time	−6.7	16	Dominant (cost saving)
Price of vaccines (for illustration only)	Both priced at 1269.5 NTD/dose	PHiD-CV reduced by 10% (1142.5 NTD/dose)	−88.2	21	Dominant (cost saving)

*base-case result: dominant/cost-saving (-6.7 million NTD with QALYs gain of 21)

*IR* incidence rate, *AOM* acute otitis media, *ICER* incremental cost-effectiveness ratio, *PCV13* 13-valent pneumococcal conjugate vaccine, *PHiD-CV* 10-valent pneumococcal polysaccharide and NT*Hi* protein D conjugate vaccine, *NTD* new Taiwan dollar, *S. pneumoniae* *Streptococcus pneumoniae*, *ST* serotype, *VT* vaccine type, *QALY* quality-adjusted life year, *yr* year

## Discussion

Evidence has suggested significant efficacy/effectiveness of PCV13 and PHiD-CV on pneumonia, IPD or meningitis hospitalization; however, no evidence could be found to demonstrate superiority of PCV13 or PHiD-CV based on their post-launch surveillance data [53]. The goal of this economic evaluation was to assess the cost-effectiveness of a PHiD-CV 2 + 1 vaccination program vs. a PCV13 2 + 1 vaccination strategy in Taiwan. This analysis incorporated a robust body of available clinical efficacy/effectiveness data for both vaccines, and local epidemiologic and cost data from the local surveillance report and published data. Price parity between vaccine costs was set to minimize the influence of price to the results and enable a clearer evaluation and comparison, which was mainly dependent on the clinical profile of the compared vaccines.

It is important to note that certain conservative assumptions were adopted in the base-case analyses for PHiD-CV. Despite independent assessments of comparable protection between the two vaccines against overall IPD and pneumonia [53, 63], a serotype-specific approach for estimating effectiveness against IPD was used (taking into account cross-protection against serotypes 6A and 19A for PHiD-CV). Furthermore, a higher effectiveness was assumed for PCV13 against pneumonia to take into account the potentially unique serotype distribution for pneumococcal pneumonia in Taiwan. The AOM-associated complications, long-term sequelae and benefits on reductions in antibiotics use were also not included in the analysis [64]. The inclusion of the above assumptions would likely result in higher cost-savings and QALYs gained due to a 2 + 1 PHiD-CV UMV as compared with a 2 + 1 PCV13 UMV.

Based on our model, both vaccines were shown to have comparable reductions in the incidence of IPD and all-cause pneumonia for a Taiwanese birth cohort over a 10-year time-horizon. However, PHiD-CV was projected to provide added benefits on AOM through protection offered against NT*Hi* AOM, which would help reduce antibiotic use and resistance among Taiwanese children. In terms of the overall financial impact, a PHiD-CV vaccination program would provide (discounted) cost-savings of 8.8 million NTD over the next 10 years per vaccinated birth cohort. Based on the scenario analysis where the cost of PHiD-CV was reduced by 10% compared to PCV13, we found that the price of vaccine was a very sensitive and significant parameter to the amount of financial savings generated by the PHiD-CV 2 + 1 vaccination program.

Extensive one-way sensitivity analysis and PSA were performed to test the results against the uncertainties of values in different parameters. In one-way sensitivity analyses, most variables did not alter the cost-saving result. Further, in a range of scenarios, PHiD-CV remained cost-saving in comparison to PCV13. The PSA found that the results were robust, with 61% certainty that PHiD-CV would be cost-saving while also generating positive health benefits.

Our findings are in line with other published cost-effectiveness analyses comparing a PHiD-CV vaccination program to PCV13. The latest publication by Shiragami et al. [65] compared PHiD-CV 3 + 1 with PCV13 3 + 1 for a paediatric UMV in Japan from both healthcare provider and societal perspectives over a time horizon of 5 years. For the Japanese birth cohort (1,042,000 newborns) the model projected that vaccination with PHiD-CV would result in cost-savings of 1.9 and 3.9 billion Japanese Yen (16 million and /33 million USD) from the healthcare provider and societal perspectives, respectively, generating an additional 433 QALYs. By et al. [66] used a Markov cohort model to compare PHiD-CV 2 + 1 and PCV13 2 + 1 strategies in Sweden from a societal perspective. It was found that the PHiD-CV strategy would generate an additional 45.3 QALYs with a substantial savings of 62 million Swedish Krona (close to 9.3 million USD) for a cohort of 112 120 children. Robberstad et al. [67] have also applied a Markov model to evaluate the cost-effectiveness of pneumococcal conjugate vaccines (PCV-7, PCV13 and PHiD-CV) for a specific birth cohort (n = 61 152) in Norway. The authors found PHiD-CV to be a dominant strategy compared with PCV13, with substantial savings of 24 million Norwegian Kroner (close to 4.15 million USD) and an increase of 49 QALYs gained. In the study published by Knerer et al. [16], it was found that PHiD-CV was again a dominant strategy as compared with PCV13, offering additional savings of 9 million Canadian Dollars (close to 9.2 million USD) in a birth cohort size of approximately 33 million newborns in Canada, and additional savings of 4.9 million British Pounds (close to 7.2 million USD) in UK with the birth cohort size of approximately 61 million newborns.

Previously published cost-effectiveness studies in the region that presented a contrasting outcome [68–71], i.e., PCV13 was a dominant option over PHiD-CV, relied on assumptions that do not hold well against the most recent body of evidence. Among these, the key assumptions were:

- Local serotype coverage-based approach for estimating vaccine effectiveness without accounting for cross-protection—a simple comparison of antigens included in the vaccine to estimate vaccine effectiveness has been shown to be erroneous in light of the evidence from well-designed studies that point to significant protection for PHiD-CV against IPD caused by serotypes 6A and 19A [13, 28, 72]. Besides, there is a growing body of evidence to show that PCV13 offers very limited to negligible protection against serotype 3 IPD [47]. These studies also extrapolated this effect to not just IPD, but all-cause pneumonia and AOM. The WHO Strategic Advisory Group of Experts (SAGE) group and Pan American Health Organization (PAHO), after examining the breadth of published evidence, stated that there was no evidence to point to the superiority of one vaccine over the other [53, 63]. This conclusion was further substantiated by a Swedish study that compared counties using PHiD-CV with those using PCV13 as a part of a UMV program; the authors also found no difference in overall IPD protection provided by either vaccine [73].
- Herd protection—some of these studies assumed no herd protection for PHiD-CV while extrapolating the herd-protection observed for PCV7 in the US to PCV13 by adjusting for the local serotype coverage. The authors have cited a lack of evidence of indirect protection for PHiD-CV as a basis for their assumption, however, recent evidence points to a significant indirect impact. One Finnish ecological study observed a 44% reduction in laboratory-confirmed IPD in unvaccinated children [74] and another demonstrated an annual decline of 2.4 and 9.2% in those aged ≥ 65 years and 18–64 years, respectively. Surveillance data from countries using PHiD-CV also clearly demonstrate VT IPD herd effects in all age groups of older adults following the introduction of childhood vaccination programs [75, 76].

As with every modeling exercise, there are a number of limitations to the current analysis. First, there are uncertainties about the herd effect in IPD due to the lack of published data on herd protection induced by each vaccine. In the current model, it was assumed that both vaccines would have the same herd protection effect, thus the inclusion or exclusion of equal herd effect would not impact the model results, unless herd protection differed by vaccine. It is difficult to accurately predict the evolution of indirect effect – these could potentially depend on the prior use of PCV7, underlying IPD serotype distribution, schedule used in children etc. [77, 78]. This assumption might be changed with the availability of future data. Another complicating feature is serotype replacement, especially in the elderly age-group [73, 76, 79]. Second, while robustly designed studies from various countries using PHiD-CV demonstrate statistically significant protection against IPD caused by serotype 19A, results from ecological studies are mixed. The potential reasons for this discrepancy are many, including confounding factors and biases inherent to such study designs. To account for this a low estimate for VE against 19A IPD was tested in a scenario analyses, and PHiD-CV still resulted in cost-savings over PCV13 indicating the importance of focusing on overall IPD protection and potential benefits against NTHi AOM from using PHiD-CV. Finally, the local data were limited as the NHIRD is a claim insurance database, which is less accurate than active surveillance data. Opinions of infectious disease specialists were used to validate the ICD-9 codes and output of incidence data to ameliorate this issue. Moreover, extensive sensitivity analyses have been conducted to test the robustness of the results and conclusion.

## Conclusion

In conclusion, PHiD-CV 2 + 1 UMV was projected to provide comparable prevention of IPD and pneumonia cases and greater reduction of AOM cases and would be cost-saving as compared with PCV13 2 + 1 in Taiwan (assuming price parity between the vaccines). This outcome was observed to hold well when accounting for parameter uncertainties using deterministic and probabilistic sensitivity analyses. The relative price of the vaccines was found to be a significant parameter that affects the results. Further studies on the indirect effect of the vaccines need to be undertaken to present a more robust result. The Additional file 2 stresses the general context and observations that were made in the present study.

## Supplementary information

**Additional file 1.** Additional Tables.

**Additional file 2.** Focus on the Patient.

## Data Availability

Data sharing not applicable to this article as no datasets were generated or analysed during the current study.

## Abbreviations

AOM: Acute otitis media
CFR: Case-fatality rate
CI: Confidence interval
GDP: Gross domestic product
NTD: New Taiwanese dollar
PSA: Probabilistic sensitivity analysis
RCT: Randomized controlled trial
TCDC: Taiwan center for disease control
TTP: Tympanostomy tube placement
UMV: Universal mass vaccination
VE: Vaccine effectiveness
VT: Vaccine-type
