# Correction to: Cost-effectiveness evaluation of the 10-valent pneumococcal non-typeable *Haemophilus influenzae* protein D conjugate vaccine for children in Taiwan

**DOI:** 10.1186/s12962-021-00284-6

**Published:** 2021-05-17

**Authors:** Chun-Yi Lu, Ching-Hu Chung, Li-Min Huang, Eliza Kruger, Seng-Chuen Tan, Xu-Hao Zhang, Nan-Chang Chiu

**Affiliations:** 1 National Taiwan University Children’s Hospital, Taipei, Taiwan; 2 Mackay Medical College, Taipei, Taiwan; 3 IQVIA Inc, Singapore, Singapore; 4 GSK, Singapore, Singapore; 5 Mackay Children’s Hospital, No. 92, Sec. 2, Zhongshan N. Rd, Taipei, 10449 Taiwan

## Correction to: Cost Eff Resour Alloc (2020) 18:30 10.1186/s12962-020-00225-9

After publication of the original article [1], the authors flagged that following an update to the Markov model during the development of the manuscript (an update which resulted in modification of the estimated savings in the Results) some out-of-date values had been erroneously retained throughout figure(s) in the article. In addition to this, the related text regarding Additional file 2 had been omitted from the end of the Conclusion section.

These errors have been corrected in the original article. Please find below a summary of the errors that have been corrected:

In the Results of the Abstract:

- “6.7 million” had been written instead of “8.8 million”
- “90.5%” had been written instead of “61%”

In Table 2, in the row ‘AOM Myringotomy’:

- “50.92%” had been written instead of “38.82%”

In the subsection ‘All-cause pneumonia effectiveness’:

- ‘23.8%” had been given for the effectiveness estimate of PCV13 against all-cause pneumonia’, while the value should read ‘23.7%’.

In Table 6, in the row ‘Death due to IPD/pneumonia’, under columns ‘PCV13 (A)’ and ‘PHiD-CV (B)’, respectively:

- “17” and “17”, respectively, had been detailed instead of “19” and “19”, respectively

In Table 6, in the row ‘All-cause pneumonia’, under the column ‘Difference (B–A)’:

- “NTD 557 957” had been written instead of “NTD 557 887”

In Table 8, column ‘Cost (millions)’ (in descending order):

− 0.6 had been written instead of − 5.9
− 6.6 had been written instead of − 8.8
− 3.9 had been written instead of − 4.8
− 0.8 had been written instead of − 1.2
− 7.1 had been written instead of − 9.3
− 14.4 had been written instead of − 19.6
- 0.15 had been written instead of 5.5
− 6.7 had been written instead of − 8.8
− 88.2 had been written instead of − 90.4

In Table 8, column ‘QALY’ (in descending order):

- 8 had been written instead of 14
- 6 had been written instead of 5
- 21 had been written instead of 22
- 38 had been written instead of 39
- 38 had been written instead of − 12

In the Footnote of Table 8:

- “− 6.7 million” had been written instead of “− 8.8 million’

At the end of the ‘Conclusion’, the following sentence was missing:

- “The supplementary Fig. 2 stresses the general context and observations that were made in the present study.”

The authors apologize for any inconvenience caused.
