# Supplementary material for: Cost-effectiveness evaluation of the 10-valent pneumococcal non-typeable Haemophilus influenzae protein D conjugate vaccine for children in Taiwan
Source: Cost Eff Resour Alloc. 2020 Aug 28;18:30. doi: 10.1186/s12962-020-00225-9 (PMC7456386; doi:10.1186/s12962-020-00225-9)
Supplement: Supplementary file 1 — Additional file 1. Additional Tables. [file 12962_2020_225_MOESM1_ESM.docx]

Cost-Effectiveness Evaluation of the 10-Valent Pneumococcal Non-typeable Haemophilus influenzae Protein D Conjugate Vaccine for children in Taiwan

Supplementary Material

**Authors:** Chun-Yi Lu^1^, Ching-Hu Chung^2^, Li-Min Huang^1^, Eliza Kruger^3^, Seng-Chuen Tan^3^, Xu-Hao Zhang^4^, Nan-Chang Chiu^2,5^

**Affiliations:**

^1^National Taiwan University Children’s Hospital, Taipei, Taiwan

^2^Mackay Medical College, Taipei, Taiwan

^3^IQVIA Inc., Singapore, Singapore

^4^GSK, Singapore, Singapore

^5^MacKay Children’s Hospital, Taipei, Taiwan

# Tables of content

[Tables 3](#_Toc10619778)

[ST1. Incidence and case fatality rate for invasive pneumococcal diseases in Taiwan 3](#_Toc10619779)

[ST2. Hospitalization rate, case fatality rate and GP consultation rate for all-cause pneumonia in Taiwan 4](#_Toc10619780)

[ST3. Hospitalization rate, case fatality rate and GP consultation rate for acute otitis media 5](#_Toc10619781)

[ST4. IPD serotype distribution in Taiwan 6](#_Toc10619782)

[Annexes 7](#_Toc10619783)

[Annex 1: Primary ICD-9codes used for NHIRD incidence and cost data analysis of all-cause pneumonia in the base-case scenario 7](#_Toc10619784)

[Annex 2: Primary ICD-9 codes used for HIRA incidence and cost data analysis of acute otitis media 7](#_Toc10619785)

[References 8](#_Toc10619786)

# Tables

## ST1. Incidence and case fatality rate for invasive pneumococcal diseases in Taiwan

| **Age group^1^** | **Incidence Rate (IR)**  **per 100,000** | **Case Fatality Rate (CFR) (%)** |
| --- | --- | --- |
| <1 year | 11.4 | 3.2% |
| 1 year | 11.4 | 3.2% |
| 2-4 years | 21.1 | 2.5% |
| 5-9 years | 1.2 | 1.3% |
| 10-19 years | 1.2 | 1.3% |
| 20-29 years | 1.1 | 7.8% |
| 30-39 years | 1.1 | 7.8% |
| 40-49 years | 1.1 | 7.8% |
| 50-59 years | 3.2 | 10.5% |
| 60-64 years | 3.2 | 10.5% |
| 65-69 years | 7.3 | 9.5% |
| 70-74 years | 7.3 | 9.5% |
| 75-79 years | 14.0 | 19.0% |
| ≥80 years | 14.0 | 19.0% |

^1^Age-specific information beyond 10 years old was provided to support the life-time analysis (alternative scenario)

Ref: [C-S Chiang, Y-Y Chen, S-F Jiang, D-P Liu, P-H Kao, H-J Teng, T-L Kuo, S-M Yao, L-R Tseng and Y-L Wang [1]](#_ENREF_1)

## ST2. Hospitalization rate, case fatality rate and GP consultation rate for all-cause pneumonia in Taiwan

| **Age group^1^** | **Hospitalization rate per 100 000^2^** | **Pneumonia case fatality rate (%)^3^** | **GP consultation rate per 100 000^2^** |
| --- | --- | --- | --- |
| <1 year | 31.06 | 0.4% | 115.10 |
| 1 year | 1242.49 | 0.1% | 6993.61 |
| 2 years | 1536.16 | 0.1% | 9650.32 |
| 3 years | 1580.28 | 0.1% | 11 006.68 |
| 4 years | 1686.03 | 0.0% | 11 888.00 |
| 5-9 years | 787.63 | 0.1% | 6222.19 |
| 10 - 14 years | 210.04 | 0.4% | 1634.45 |
| 15 - 19 years | 117.86 | 1.4% | 936.10 |
| 20 - 24 years | 98.79 | 1.0% | 772.29 |
| 25 - 29 years | 115.73 | 2.1% | 1006.32 |
| 30 - 34 years | 146.10 | 3.2% | 1342.97 |
| 35 - 39 years | 201.13 | 4.7% | 1618.55 |
| 40 - 44 years | 258.87 | 6.6% | 1592.96 |
| 45 - 49 years | 348.65 | 7.5% | 1676.84 |
| 50 - 54 years | 497.13 | 7.2% | 2162.05 |
| 55 - 59 years | 726.40 | 7.2% | 2893.85 |
| 60 - 64 years | 1145.43 | 6.6% | 4015.74 |
| 65 - 69 years | 1895.44 | 6.7% | 5713.60 |
| 70 - 74 years | 3112.94 | 8.2% | 8235.38 |
| 75 - 79 years | 5548.83 | 9.9% | 12 652.44 |
| 80 - 84 years | 10 085.95 | 11.2% | 20 190.93 |
| 85 - 89 years | 16 264.84 | 12.3% | 29 352.94 |
| 90+ | 25 303.73 | 13.7% | 43 899.90 |

^1^ Age-specific information beyond 10 years old was provided to support the life-time analysis (alternative scenario)

^2^ Based on 2012 NHIRD entire population data

^3^ Unpublished data [[2](#_ENREF_2)]

ST3. Hospitalization rate, case fatality rate and GP consultation rate for acute otitis media

| **Age group^1^** | **GP consultation rate per 100 000** | **Hospitalization rate per 100 000** |
| --- | --- | --- |
| <1 year | 8954.4 | 11.9 |
| 1 year | 10 738.0 | 1011.3 |
| 2 years | 10 738.0 | 1026.1 |
| 3 years | 10 738.0 | 826.2 |
| 4 years | 10 738.0 | 795.8 |
| 5-9 years | 2888.6 | 245.8 |
| 10-14 years | 2888.6 | 37.7 |
| 15-19 years | 865.8 | 14.1 |
| 20-24 years | 865.8 | 7.8 |
| 25-29 years | 689.0 | 11.8 |
| 30-34 years | 689.0 | 16.4 |
| 35-39 years | 689.0 | 18.9 |
| 40-44 years | 689.0 | 25.6 |
| 45-49 years | 488.8 | 41.3 |
| 50-54 years | 488.8 | 46.9 |
| 55-59 years | 488.8 | 50.4 |
| 60-64 years | 488.8 | 65.8 |
| 65-69 years | 397.8 | 63.3 |
| 70-74 years | 397.8 | 63.4 |
| 75-79 years | 265.2 | 57.7 |
| 80-84 years | 265.2 | 47.0 |
| 85-89 years | 265.2 | 47.8 |
| 90+ | 265.2 | 55.8 |

^1^: Age-specific information beyond 10 years old was provided to support the life-time analysis (alternative scenario)

Ref: Based on the 2012 NHIRD entire population data

## ST4. IPD serotype distribution in Taiwan

|  | Age | | | |
| --- | --- | --- | --- | --- |
| Serotype | **0 to <2^1^** | **2 to <5^1^** | **5 to <10^2^** | **10+^2^** |
| 1 | 0.00% | 0.00% | 0.0% | 0.0% |
| 3 | 3.75% | 0.02% | 12.8% | 12.8% |
| 4 | 0.00% | 0.00% | 0.5% | 0.5% |
| 5 | 0.00% | 0.00% | 0.0% | 0.0% |
| 6A | 8.75% | 3.89% | 3.5% | 3.5% |
| 6B | 5.00% | 4.72% | 8.5% | 8.5% |
| 7F | 0.00% | 0.00% | 0.0% | 0.0% |
| 9V | 0.00% | 0.00% | 1.9% | 1.9% |
| 14 | 7.50% | 5.56% | 16.3% | 16.3% |
| 18C | 0.00% | 0.00% | 0.0% | 0.0% |
| 19A | 41.25% | 56.39% | 14.9% | 14.9% |
| 19F | 8.75% | 8.89% | 8.6% | 8.6% |
| 23F | 7.50% | 5.83% | 11.7% | 11.7% |
| Other | 17.50% | 14.66% | 21.2% | 21.2% |

^1^ [S-H Wei, C-S Chiang, C-H Chiu, P Chou and T-Y Lin [3]](#_ENREF_3)

^2^ [C-S Chiang, Y-Y Chen, S-F Jiang, D-P Liu, P-H Kao, H-J Teng, T-L Kuo, S-M Yao, L-R Tseng and Y-L Wang [1]](#_ENREF_1)

# Annexes

## Annex 1: Primary ICD-9codes used for NHIRD incidence and cost data analysis of all-cause pneumonia in the base-case scenario

| Code | Description |
| --- | --- |
| 481 | Pneumococcal pneumonia [*Streptococcus pneumoniae* pneumonia] |
| 482.2 | Pneumonia due to *Hemophilus influenzae* [*H. influenzae*] |
| 482.9 | Bacterial pneumonia, unspecified |
| 486 | Pneumonia, organism unspecified |

## Annex 2: Primary ICD-9 codes used for HIRA incidence and cost data analysis of acute otitis media

| Code | Description |
| --- | --- |
| 382.00 | Acute suppurative otitis media without spontaneous rupture of ear drum |
| 382.01 | Acute suppurative otitis media with spontaneous rupture of ear drum |
| 382.4 | Unspecified suppurative otitis media |
| 382.9 | Unspecified otitis media |

# References

1. Chiang C-S, Chen Y-Y, Jiang S-F, Liu D-P, Kao P-H, Teng H-J, Kuo T-L, Yao S-M, Tseng L-R, Wang Y-L: **National surveillance of invasive pneumococcal diseases in Taiwan, 2008–2012: Differential temporal emergence of serotype 19A**. *Vaccine* 2014, **32**(27):3345-3349.

2. Chang et al: **Burden of pneumococcal diseases, acute otitis media and an economic analysis of pneumococcal vaccines in Taiwan**. In*.* Edited by Unpublished data; 2010.

3. Wei S-H, Chiang C-S, Chiu C-H, Chou P, Lin T-Y: **Pediatric Invasive Pneumococcal Disease in Taiwan Following a National Catch-up Program With the 13-Valent Pneumococcal Conjugate Vaccine**. *The Pediatric infectious disease journal* 2015, **34**(3):e71-e77.
