# Supplementary material for: Cost-effectiveness evaluation of the 10-valent pneumococcal non-typeable Haemophilus influenzae protein D conjugate vaccine for children in Taiwan
Source: Cost Eff Resour Alloc. 2020 Aug 28;18:30. doi: 10.1186/s12962-020-00225-9 (PMC7456386; doi:10.1186/s12962-020-00225-9)
Supplement: Supplementary file 2 — Additional file 2. Focus on the Patient. [file 12962_2020_225_MOESM2_ESM.docx]

# Focus on the Patient

**What is the context?**

- *Streptococcus pneumoniae* and *Haemophilus influenzae (Hi)* bacteria are major causes of infections such as invasive pneumococcal diseases, pneumonia and acute otitis media.
- In Taiwan, two vaccines against these infections are licensed for children: 10-valent pneumococcal polysaccharide and NT*Hi* protein D conjugate vaccine (PHiD-CV; Brand name: Synflorix) and 13-valent pneumococcal conjugate vaccine (PCV13, Brand name: Prevenar 13).
- The government implemented PCV13 as part of the universal mass vaccination program for young children in 2015. It requires an updated cost-effectiveness analysis to inform its decision about which vaccine to implement on the upcoming universal mass vaccination program.

**What is new?**

- We evaluate how cost-effective it would be to use PHiD-CV in the Taiwan universal mass vaccination program compared with PCV13 which is currently used. The analysis took into consideration the latest data on vaccine effectiveness and local data on disease prevalence and costs.
- Our analysis indicates that PHiD-CV is cost-saving, can provide similar protection against invasive pneumococcal diseases and achieves better reduction in acute otitis media (AOM) cases compared with PCV13.

**What is the impact?**

- Given the substantial burden of *S. pneumoniae*- and Non-Typeable *Hi* (NTHi)-related diseases, this revised economic analysis will allow decision-makers in Taiwan to make an informed choice when implementing their universal mass vaccination program.
